# Quantitative *Ex-Vivo* Micro-Computed Tomographic Imaging of Blood Vessels and Necrotic Regions within Tumors

**DOI:** 10.1371/journal.pone.0041685

**Published:** 2012-07-25

**Authors:** Charlene M. Downey, Arvind K. Singla, Michelle L. Villemaire, Helen R. Buie, Steven K. Boyd, Frank R. Jirik

**Affiliations:** 1 Department of Biochemistry and Molecular Biology, University of Calgary, Calgary, Alberta, Canada; 2 Department of Mechanical and Manufacturing Engineering, University of Calgary, Calgary, Alberta, Canada; 3 The McCaig Institute for Bone and Joint Health, University of Calgary, Calgary, Alberta, Canada; Virginia Tech, United States of America

## Abstract

Techniques for visualizing and quantifying the microvasculature of tumors are essential not only for studying angiogenic processes but also for monitoring the effects of anti-angiogenic treatments. Given the relatively limited information that can be gleaned from conventional 2-D histological analyses, there has been considerable interest in methods that enable the 3-D assessment of the vasculature. To this end, we employed a polymerizing intravascular contrast medium (Microfil) and micro-computed tomography (micro-CT) in combination with a maximal spheres direct 3-D analysis method to visualize and quantify *ex-vivo* vessel structural features, and to define regions of hypoperfusion within tumors that would be indicative of necrosis. Employing these techniques we quantified the effects of a vascular disrupting agent on the tumor vasculature. The methods described herein for quantifying whole tumor vascularity represent a significant advance in the 3-D study of tumor angiogenesis and evaluation of novel therapeutics, and will also find potential application in other fields where quantification of blood vessel structure and necrosis are important outcome parameters.

## Introduction

By providing oxygen and nutrients, as well as a route for metastatic spread, angiogenesis is essential for the growth and progression of solid tumors [1]–[3]. This has stimulated the development and application of anti-angiogenenic therapeutics such as bevicuzimab, a monoclonal antibody that targets VEGF-A, a key growth factor required by newly developing blood vessels [4], as well as vascular disrupting agents (VDA), such as 5,6-dimethylxanthenone-4-acetic acid (DMXAA), that can cause the rapid and relatively selective disruption of the tumor vasculature [2], [3].

At present, relatively little information is available about the 3-D organization of the tumor vasculature and how these structures respond to treatment with anti-angiogenic agents. Previously, tumor vessel imaging has relied upon labour intensive 2-D methodologies such as serial immunohistological sectioning or confocal microscopy to reconstruct the vascularity of small regions, however, these methods fail to fully capture the complexity of vessel trajectories and organization throughout the entire tissue. To address this, several novel techniques have been developed for 3-D imaging of vasculature both in vivo and ex-vivo. While in vivo imaging has the benefit of being able to follow vessel growth and modifications in real time, full 3-D quantification at high resolution of the entire tumor is better achieved using ex-vivo analyses. For example, in vivo methods such as contrast-enhanced ultrasound imaging or magnetic resonance imaging (MRI) can yield volumetric evaluation of vessel volume and blood perfusion, yet with very low resolution of 3-D vascular structures [5], [6]. In addition, although intravital multi-photon microscopy can achieve in vivo imaging of vessels at high resolution, being limited in terms of the depth of the tissue region that can be analyzed (250–400 µm) [7], it is unable to provide a global assessment of vascular structure.

Ex-vivo 3-D vascular analysis on the other hand, such as scanning electron microscopy (SEM), optical coherence tomography (OCT), optical projection tomography (OPT), or the use of radio-opaque silicon polymers such as Microfil (MV-122) in combination with micro-CT, provide a novel approach to imaging the entire tumor vasculature in 3-D [7]–[16]. These methods have previously been used to evaluate the angiogenic effects of specific cytokines [17], the anti-angiogenic effects of VEGF-blocking antibodies [4], [18], [19], and for monitoring vessel development and patterning [1], [15], [16], [20], [21]. They have also permitted the imaging of tumor vessels within mice (**Figure S1**).

In this study, we have combined an advanced bone morphometric analysis method, the maximal spheres algorithm, with the use of an established 3-D ex-vivo vascular imaging method (Microfil in combination with micro-CT), creating a novel way to characterize blood vessel microarchitecture and regions of necrosis within subcutaneous tumors. Conventional 3-D analytical techniques use multiple indirect 2-D measurements to generate an assumed 3-D structure based on a structure model algorithm [22], [23], however, increased variability or randomness within a sample increases the errors within these 3-D assumptions. The introduction of the maximal spheres algorithm allows for direct measurement of 3-D objects, without any requirement for a structure model assumption, by calculating a direct volume based on fitting maximal spheres to every point within the structure. From these calculations, the actual volume distributions of spheres within any sample can be obtained [22], [23]. The tumors that were analyzed with these computational methods were as follows: the human non-small cell lung cancer (NSCLC) line, H1299; the human breast cancer line, MDA-MB-231; and two murine NSCLC cell lines, 344SQ and 7417PF, isolated from metastatic lung carcinomas arising in mice harbouring *p53^R172HΔg/+^* and *Kras^LA1/+^* mutations [24], [25].

## Materials and Methods

### Ethics Statement

All animal studies were approved by the University of Calgary Animal Care Committee under protocol number M10063.

### Cell Lines and Culture

The human NSCLC cell line H1299 was obtained from ATCC (CRL-5803: NCI-H1299) and the human breast adenocarcinoma cell line MDA-MB-231 (ATCC: HTB-26) was kindly provided by Dr. T. Guise (University of Virginia) [26]. Murine lung cancer cell lines were generated from lung tumors and metastases arising in *p53^R172HΔg/+^ Kras^LA1/+^* mice as previously described [25]. The 344SQ cell line was kindly provided by Dr. J. Kurie (University of Texas, MD Anderson) [25] and the 7417PF cell line was generated from a peritoneal fat pad metastasis from the same strain of mice as per ethics protocol M010063 from the University of Calgary Animal Care Committee. Cells were cultured in RPMI 1640 (NSCLC) or DMEM (Invitrogen) supplemented with 10% fetal bovine serum (FBS), 100 U ml^–1^ penicillin, 100 mg ml^–1^ streptomycin at 37°C in a 5% CO_2_ humidified atmosphere, and routinely passaged every 2–3 d. All cell lines were confirmed to be free of pathogenic murine viruses and *Mycoplasma spp.* by PCR testing at Charles River Laboratories (Wilmington, MA).

The 344SQ and MDA-MB-231 cells were stably transfected with the dual-reporter pEGFP-Luc2 vector using the Lipofectamine reagent (Invitrogen) as previously described [27]. Transfectants were selected in media containing 0.8 mg ml^–1^ geneticin (Invitrogen) and fluorescence-activated cell sorting (FACS) (Flow Cytometry Core Facility, University of Calgary) was used to isolate the polyclonal cell population expressing the highest EGFP fluorescence signals.

### Tumor Generation

Nude-beige (NIH-III) mice (6–8 week old) were purchased from Charles River Laboratories, and wild-type 129/Sv mice (6–8 week old) from our colony were used. Mice were maintained on standard mouse chow (Pico-Vac Lab Mouse Diet #5062), and housed in a barrier facility in accordance with both University of Calgary Animal Care Committee, and Canadian Council on Animal Care guidelines. Single cell suspensions of 5×10^5^ cells in 100 µl PBS from the four different cell lines were injected subcutaneously over both the right and left posterior flank of the NIH-III mice (N = 3–4 mice per cell line). Tumor growth was monitored regularly with calliper measurements until tumors reached the ∼1 cm diameter end-point. Similarly, subcutaneous injections of 5×10^5^ cells in 100 µl PBS of 344SQ-EGFP-Luc2 (344SQ-EL) in wild-type 129/Sv mice (N = 10 mice), and 1.2×10^6^ cells in 100 µl PBS of MDA-MB-231-EGFP-Luc2 in NIH-III mice (N = 3 mice) were performed. Tumor growth was monitored every 3–4 days via bioluminescence imaging (BLI) (IVIS-Lumina, Caliper Life Sciences) as previously described [27]. To avoid light signal attenuation due to fur, 129/Sv mice were shaved over the tumor injection site prior to imaging. On day 13 (344SQ-EL) or 30 (MDA-MB-231-Luc2) post-tumor cell inoculation, mice were administered 25 mg kg^–1^ of 5,6-dimethylxanthenone-4-acetic acid (DMXAA; D5817, Sigma-Aldrich), or vehicle control (dimethylsulfoxide) via i.p. injection and imaged again via BLI at 6 and 24 hrs prior to sacrifice [11].

### Vascular Perfusion

Prior to sacrifice, mice were given a sub-lethal i.p. dose of ketamine (100 mg kg^–1^) plus xylazine (6 mg kg^–1^). Vascular perfusions were carried out as previously described [20]. The abdominal cavity was opened, the diaphragm was incised to expose the pleural cavity, and the ribs were cut away to allow access to the heart. A 25G butterfly needle attached to 0.75 in polyethylene tubing (BD Vacutainer) was inserted into the left ventricle and the right jugular vein severed to provide a drainage point. The circulatory system was flushed with 10 ml of heparinised saline, and this was immediately followed by perfusion with 10 ml of radio-opaque silicone rubber polymer (Microfil®, MW-122 yellow, Flow-Tech) at a constant rate of 0.75 ml min^–1^ using a Harvard Apparatus PHD 2000 Infusion pump (Instech Laboratories Inc). The Microfil compound was allowed to polymerize overnight at 4°C. Tumors, and kidneys were excised and fixed in 10% neutral buffered formalin (NBF) for a minimum of 24 hrs prior to micro-CT scanning.

### Micro-computed Tomography

Tumors were scanned using a µCT35 instrument (Scanco Medical AG) at a nominal resolution of 10 µm (55 kVp, 145 µA, 200 ms integration time, 1000 proj/180 degrees, 20.5 mm diameter field of view, 2048×2048 reconstruction matrix, conebeam reconstruction). Gaussian smoothing and global thresholding procedures were applied to the grayscale data to contour the tumor (sigma = 2, support = 3, threshold = 30% of max grayscale value) and extract the tumor vascular network (sigma = 0.8, support = 1, threshold = 200% of max grayscale value). The resulting segmented images were analyzed using direct 3-D measurement techniques, voxel-counting and maximal spheres [22], to quantify the tumor volume, as well as vessel volume, density, thickness, and separation.

### Histopathology

H1299 tumors from NIH-III mice and 344SQ-EL tumors from 129/Sv mice receiving DMXAA or vehicle control were fixed in 10% NBF and were paraffin embedded and sectioned into 4 µm sections. Representative sections were stained with H&E or an antibody to alpha-smooth muscle actin (α-SMA) (abcam) in combination with the Vectastain Rabbit ABC Kit (Vector Labs), and counterstained with haematoxylin. CD31 staining (abcam) was performed on OCT embedded frozen sections from H1299 tumors.

### Statistics

Where applicable, average data was compared with a student’s *t-*test, with Welch’s correction on samples with unequal variance. Histogram data was compared with a one-way ANOVA with Bonferroni’s post test between all samples. *P* values of <0.05 were considered significant.

## Results

**Figure 1:**
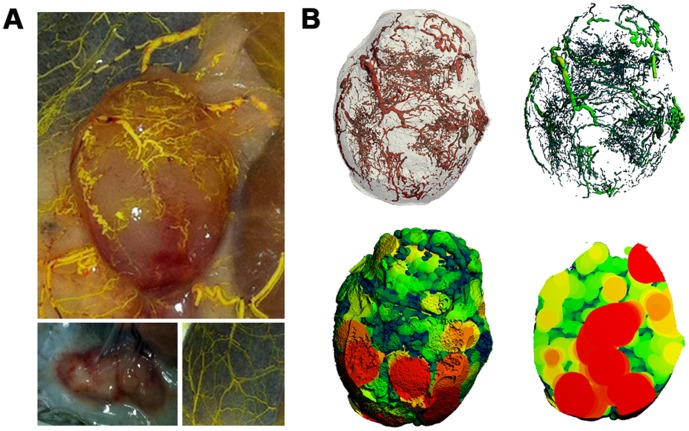
Microfil combined with micro-CT. (**A**) Image of a Microfil-perfused tumor grown subcutaneously in a mouse (top). The Microfil polymer is yellow, allowing one to visualize the effectiveness of the perfusion. Images of a non-perfused tumor (bottom left), and of the perfused dermis without a tumor (bottom right) are included as a control. (**B**) 3-D reconstructions of the tumor in (**A**) showing a surface rendering of the vasculature (top left), the thickness of the vessels demonstrated by a heat map and the intravascular distances (top right), or vessel separation demonstrated with a sphere-filling model (bottom left – full view, bottom right – cross-section through centre of tumor). Note that the vessels are not shown in the sphere-filled images, only the inter-vessel spaces.

To characterize the vasculature of the different subcutaneously grown cell lines, mice were terminally perfused with Microfil (**Figure 1A**) and whole tumors were subjected to micro-CT imaging and analysis. Through application of traditional bone morphometric analyses, we were able to not only re-create a 3-D reconstruction of the Microfil-perfused vessels, but we were also able to generate novel images allowing measurements of vessel thickness and intravascular distances (**Figure 1B**). The latter providing a novel method of imaging vessels, and importantly, a way to quantify areas of hypoperfusion and necrosis in 3-D.

### Three-dimensional Reconstruction and Quantification of Vascular Network

**Figure 2:**
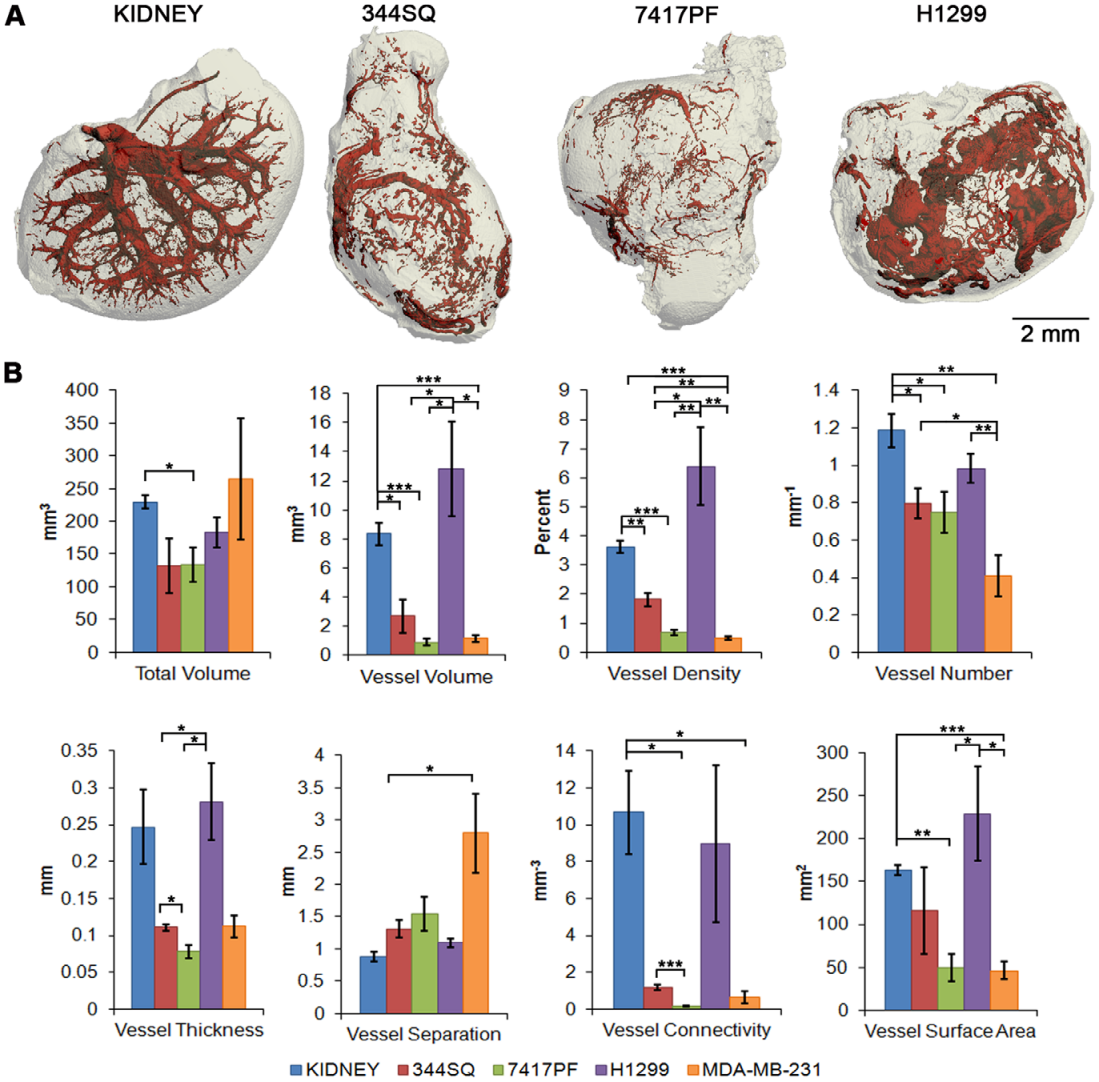
3-D renderings and quantification of tumor vasculature. (**A**) 3-D micro-CT surface renderings of a Microfil-perfused kidney (left) and three subcutaneous tumors derived from NSCLC cell lines: 344SQ (2^nd^ from left), 7417PF (3^rd^ from left) and H1299 (right). The entire tissue or tumor mass is in gray, and the vasculature in red. Using this imaging analysis in combination with 3-D measurement techniques, voxel-counting and maximal spheres analysis, two murine NSCLC cell lines (344SQ and 7417PF), and two human cell lines (the NSCLC cell line H1299 and the breast cancer line, MDA-MB-231) were examined. For comparison purposes, the kidney, being similar in size to the subcutaneous tumors, was also examined. (**B**) Quantification yielded representative average measurements for total volume (TV), vessel volume (VV), vessel density (VV/TV), vessel number (V.N), vessel thickness (V.Th), vessel separation (V.Sp), vessel connectivity or branching (V.ConnD), and vessel surface area (V.SA). Data are shown as the average ± s.e.m. (N = 3–6 per cell line). All of the cell lines (apart from H1299) demonstrated significant variability in vessel parameters compared with the kidney. Asterisks indicate: * p<0.05, ** p<0.01, *** p<0.001.

Comparable to previous studies [1], [4], [17]–[19], [28], surface renderings generated from the representative tumors (gray) demonstrated a poorly organized vascularity (red) as compared to the structurally well-organized vasculature of the kidney (**Figure 2A** and **Movie S1**). The disorganized tumor vasculature demonstrated a relatively chaotic pattern, with blunt ends, uneven widths, and areas not filled by contrast agent. The latter were suggestive of ischemic or necrotic areas within the tumors.

In order to characterize the vascular differences amongst the tumors, binarized images were quantified with standard average 3-D measurements with respect total volume (TV), as well as vessel volume (VV), vessel density or partial tumor volume (VV/TV), vessel number (V.N), vessel thickness (V.Th), vessel separation (V.Sp), vessel connectivity density (V.ConnD) and vessel surface area (V.SA) (**Figure 2B**). Previous studies of vascularity have utilized TV, VV, VV/TV and V.Th in their analysis [1], [4], [17], [18], [28], [29], and we have added V.N, V.ConnD, V.SA, and V.Sp, the latter providing a potential means for quantifying necrotic volumes in 3-D. These morphometric measures are akin to the measures used to quantify bone morphology [14], [30]. While there was no significant difference in average total volume between the cell lines or kidney (apart from 7417PF), we found considerable variability in VV, VV/TV and V.N (**Figure 2B**). Interestingly, quantification of the H1299 vasculature using these parameters failed to demonstrate any difference from the renal vasculature, however, upon 3-D visualization the tumor vasculature (**Figure 2A**) this similarity was revealed to result from large bulbous spaces in H1299 tumors that had become filled with contrast agent. The other tumors did not show these dilated structures. It was notable that the two murine cell lines (344SQ and 7417PF), despite originating from mice with the same genetic background, demonstrated significantly different V.Th and V.ConnD, thus providing evidence of intra-tumoral heterogeneity with respect to angiogenic processes.

While the measurements described above allow for 3-D quantification of vascular parameters, these measurements are restricted to an average of the vasculature across an entire tumor, and cannot extrapolate changes happening in one area of a tumor over another. Using the maximal sphere-filling model [22], [23], however, we can calculate the volume of spheres at increasing diameter ranges for both vessel thickness (V.Th) and vessel separation (V.Sp) (measuring the thickness of the tumor tissue lying between vessels), a novel use of this method and a means of quantifying avascular regions within a tumor. By applying the data over a histogram, we can clearly see the sphere diameter ranges that are present within a given tumor.

**Figure 3:**
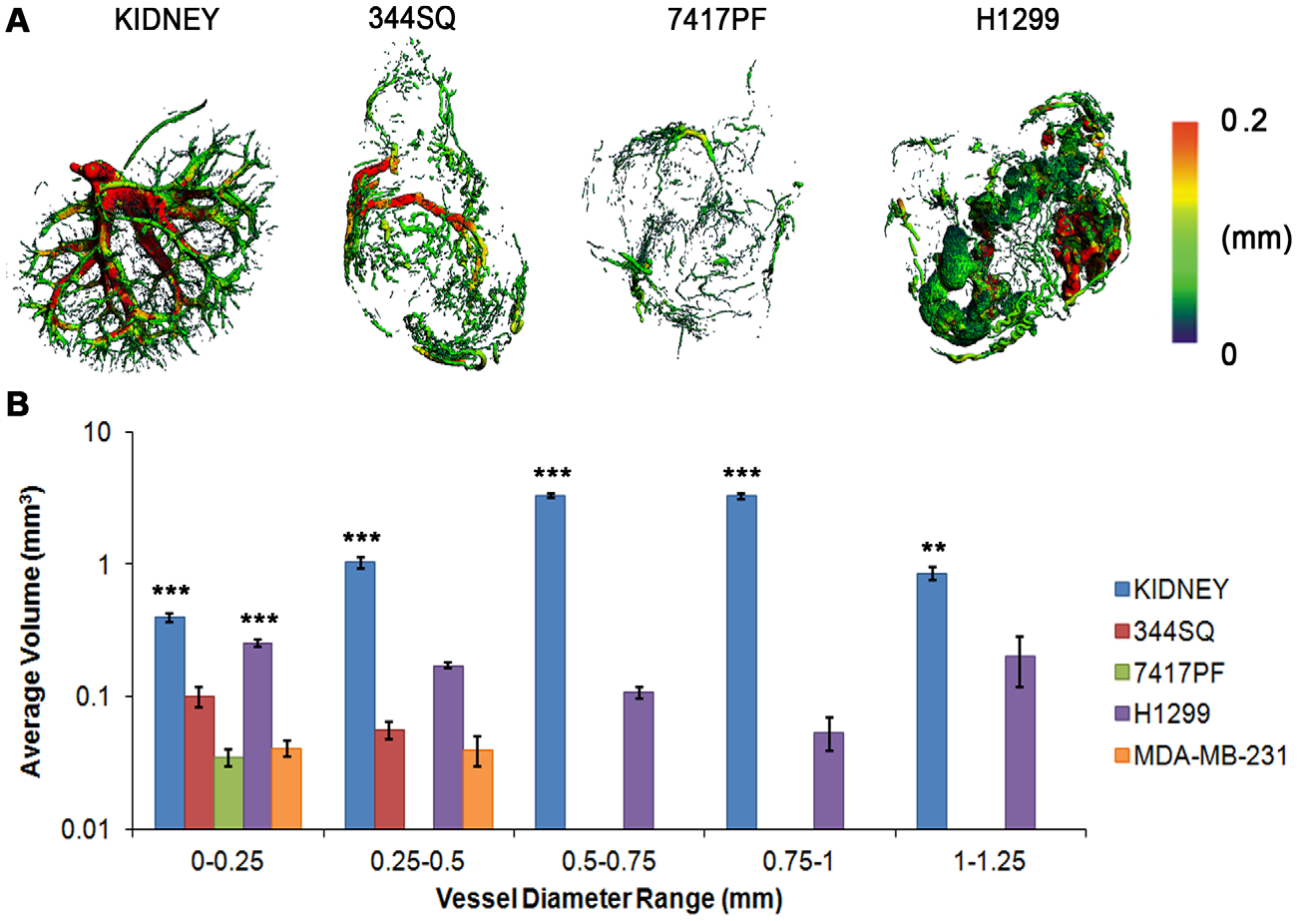
Quantification of vessel thickness. (**A**) 3-D renderings of vasculature (red  =  vessel diameters of 0.2 mm or greater) showing the organized kidney vasculature versus the disorganized, irregular vessel diameters in the NSCLC tumors (344SQ, 7417PF and H1299). (**B**) Quantification of vessel thickness of two human and two murine cell lines over the entire spectrum of vessel diameters, showing very significant differences between normal and tumor vasculature, with most of the tumors only having vessels up to 0.5 mm, while the kidneys had vessels up to 1.25 mm in diameter (note the log scale on Y axis).

Vessel thickness (V.Th), for example, indicated with a heat map where vessel diameters of 0.2 mm or greater are represented in red (**Figure 3A** **and Movie S2**), also demonstrates abnormalities of vessel structure within the tumors as compared to the renal vasculature. While there was no statistical significance in average V.Th between the kidney and the tumors **(** **Figure 2B**), quantification of the average volume of vessels present at any given diameter demonstrated a dramatic difference between them (**Figure 3B**). The kidney vasculature diameters followed a Gaussian distribution, while the tumor vasculature did not exceed sizes of 0.25 or 0.5 mm (7417PF and 344SQ/MDA-MB-231 respectively). As previously noted, the H1299 tumors exhibited large bulges filled with Microfil extending up to 1.25 mm in diameter. Extravasation of Microfil from leaky tumor vessels could potentially explain this phenomenon, however, it is unlikely that such large bulges would occur due to Microfil leakage alone, since this would involve considerable tissue compression. Alternatively, another study has reported small globular spheres on Microfil-perfused vessels, determined to be indicative of flow obstruction due to vascular endothelial cell endocytosis of the heavy metal within Microfil necessary for X-ray contrast [9], however, bulges as large as the ones seen in our tumor cell lines have not been reported. Upon histological analysis of non-Microfil perfused H1299 tumors **(Figure S2)**, large empty necrotic areas were observed in close proximity to tumor vessels. Filling of these spaces with Microfil would readily account for the large globular structures seen in the micro-CT images. These results emphasize the need to not rely solely upon the quantitative parameters to assess the vasculature, but to also take into consideration the 3-D images of the structure being analyzed as well as the histopathology of a given tumor type (**Figure 2A**).

### Quantification of Avascular Hypoxic Areas

**Figure 4:**
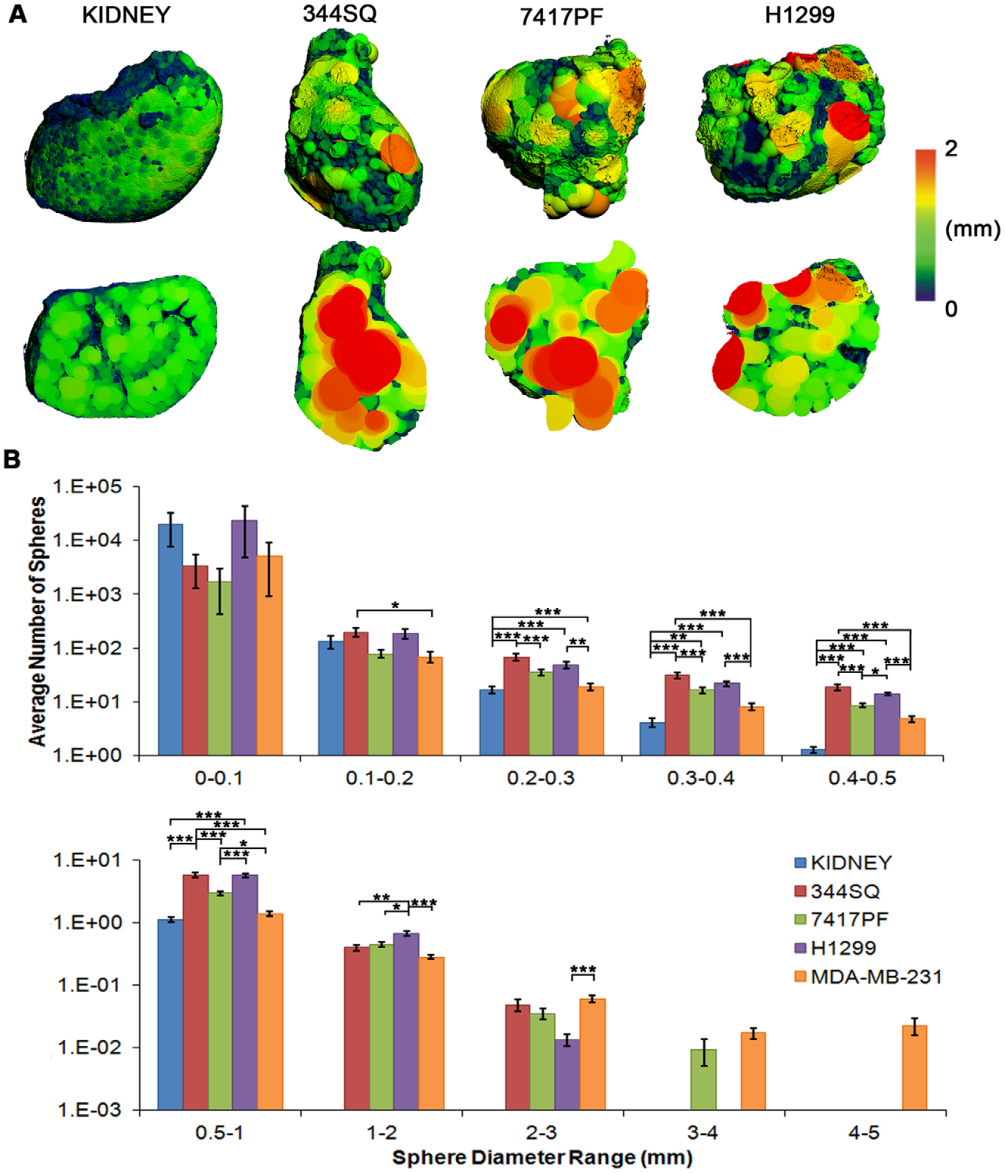
Imaging and quantification of avascular necrosis. (**A**) 3-D renderings from the kidney and NSCLC tumors following application of the maximal sphere-filling model; intact sample views above and cross-sectional images below (red indicating 2 mm or greater diameter of sphere). The presence of red (avascular) areas can be seen in all the tumor cell lines tested. (**B**) Histogram quantification of the maximal-spheres of two human and two murine cell lines by evaluating the total number of spheres present at increasing ranges of diameters (from 10 µm to 5 mm) (note the log scale on Y axis). Considerable variability is evident within both the vascularised and avascular areas within the tumors, with significantly more avascular areas being present in tumors, in contrast to the well-vascularized kidneys. Data indicate averages ± s.e.m. (N = 3–6 tumors per cell line). Asterisks indicate: * p<0.05, ** p<0.01, *** p<0.001.

While the relatively empty, resolving areas of necrosis in the slow growing H1299 tumors (**Figure S2**) became filled with contrast agent, the more rapidly growing murine NSCLC line tumors showing fresh areas of necrosis (**Figure S3**), were indicated by regions that were not perfused by Microfil (**Figure 2A**). Necrotic volume is an important variable that is not generally considered during conventional micrometer-based measurements of subcutaneous tumor growth. Thus, to quantify avascular areas in the NSCLC and the MDA-MB-231 tumors, we employed measurements of vessel separation (V.Sp) using a sphere-filling model [22], [23] (red indicating 2 mm diameter or greater) (**Figure 4A**), a novel application of this method. These separation images are representative surface renderings and cross-sectional images taken through the centre of the tumor (**Figure 4A**), however, visualization of avascular regions is best carried out by scans through the entire tumor (**Figure S4** and **Movie S3**). Through quantification of histogram data, the number of spheres present at different diameters (**Figure 4B**) clearly reveals the presence of large avascular regions among all the tumor cell lines (indicated by a 1 mm or greater separation between vessels), that are absent in the kidney. In addition, there were significant differences between the kidney and the tumors with respect to the smaller separation diameters: the latter being indicative of well-vascularized areas. Interestingly, similar to the other tumors, the H1299 tumors in addition to having bulging contrast-filled regions (max V.Th  = 1.25 mm), also contained large avascular areas (max V.Sp  = 2.94 mm), perhaps indicative of necrotic areas lacking nearby competent vessels.

### Vascular Disrupting Agent Drug Validation

**Figure 5:**
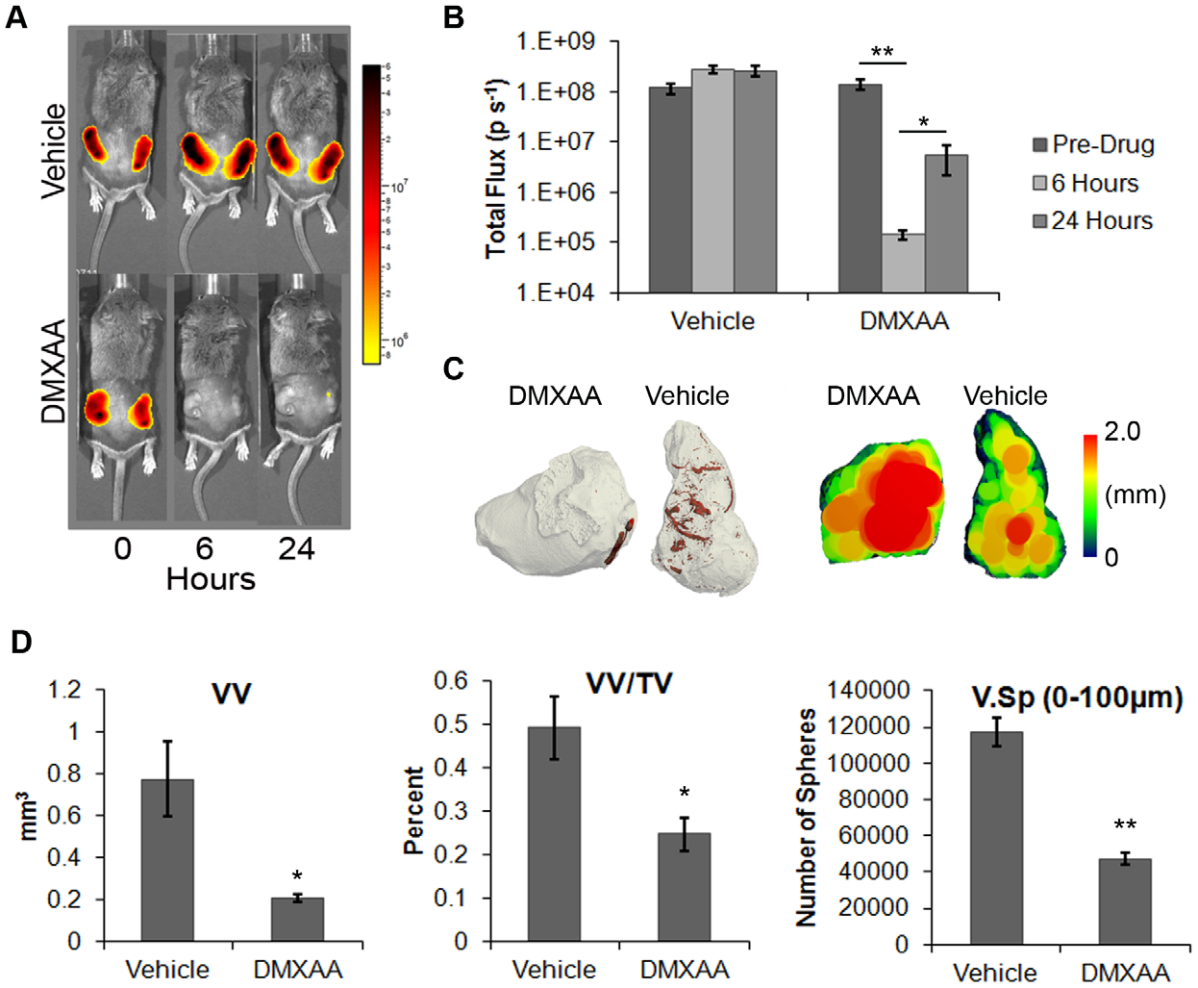
Assessing the effects of a vascular disrupting agent. (**A**) Bioluminescence images of luciferase-expressing 344SQ-EL cell line-derived subcutaneous tumors. Images were taken prior to treatment, and again at 6 and 24 hrs post DMXAA treatment. This agent led to a dramatic loss of bioluminescence. (**B**) Quantification of changes in photon emission rates of the DMXAA-treated, and control tumors (data represent averages ± s.e.m., N = 20). (**C**) 3-D micro-CT surface renderings (gray and red) and cross-sectional maximal-spheres filling model rendering (red  =  vessel diameters of 2 mm or greater) for representative subcutaneous tumors treated with either DMXAA or vehicle control, and perfused with Microfil after 24 hrs. The images demonstrate a large increase in the necrotic regions following DMXAA treatment. (**D**) Quantification of the vessel volume, density, and separation of DMXAA-treated tumors. There was a decrease in vessel volume and density, and in well-vascularized areas, as indicated by the drop in spheres present with sizes between 0–100 µm. This result was consistent with an increase in the size of the necrotic areas in the DMXAA-treated tumors. Data indicate averages ± s.e.m., N = 6 per sample. Asterisks indicate: * p<0.05, ** p<0.01.

In order to determine the potential of this 3-D imaging technique for quantifying changes brought upon by a VDA, mice bearing luciferase-expressing (344SQ-EL) subcutaneous tumors were treated with DMXAA or vehicle control, and the tumors were evaluated after 6 and 24 hours. DMXAA acts by selectively inducing damage and apoptosis of tumor vascular endothelial cells leading to measurable reductions in blood flow within 30 minutes, and becomes maximal by 6–24 hours as a result of vessel thrombosis [2], [3]. In vivo bioluminescence imaging of luciferase-expressing tumors at both 6 and 24 hours after DMXAA revealed a dramatic loss of signal (**Figure 5A**) owing to dramatic decreases (1,000-fold, p = 0.003) in photon emission rates in the DMXAA treated mice (**Figure 5B**). At necropsy, DMXAA-treated tumors were hemorrhagic, and this was confirmed by histological analysis, that showed vessel thrombosis and hemorrhage in the tumor periphery (**Figure S5**). These gross and histopathological effects of DMXAA are consistent with previous reports [2], [3], [31]. Notably, whereas quantifying the effects of a drug on the entire 3-D vascular network of a tumor via immunohistochemistry alone is difficult, the combined approach of Microfil and micro-CT simplifies the process considerably.

Applying the methods of analysis described above to DMXAA-treated NSCLC tumors, we found a reduction in contrast medium filling of tumor vessels in the DMXAA-treated mice (**Figure 5C**), that was accompanied by a significant drop in VV and VV/TV (p = 0.05 and p = 0.03, respectively) (**Figure 5D**). Representative cross-sectional images of V.Sp (**Figure 5C**) demonstrated a large increase in central tumor necrosis in the DMXAA treated mice, and a significant decrease of well-vascularized areas as indicated by a drop in the number of spheres present sized between 0–100 µm diameter using the V.Sp maximal-spheres model (p = 0.005) (**Figure 5D**). These results were indicative of an increase in avascular areas in DMXAA treated tumors. In contrast to the response of the tumors, DMXAA had no significant effect on the renal vessel parameters (**Figure S6**). Other studies using similar 3-D analysis techniques needed to exclude samples with large areas of necrosis from their analysis since these led to errors in their measurements [28]. However, we believe that information about necrotic regions, as provided by the maximal-spheres method, will be a critical component in the evaluation of drugs targeting the tumor vasculature or the tumor itself.

**Figure 6:**
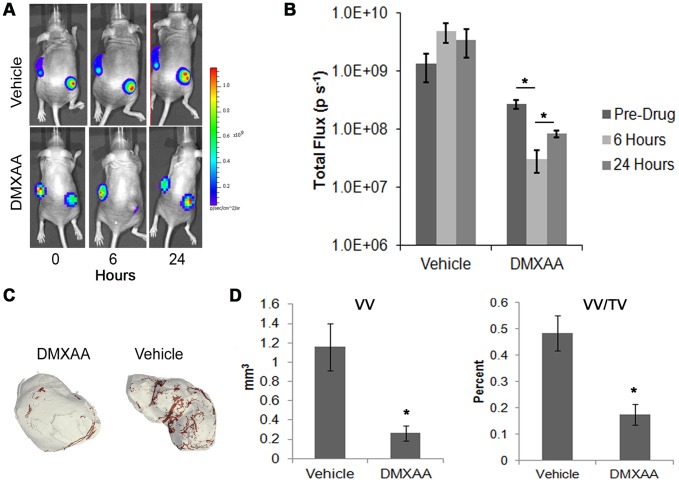
Validation of VDA with a human cell line. (**A**) Bioluminescence images taken just prior to DMXAA treatment, as well as at 6 and 24 hrs post treatment of NIH-III mice bearing subcutaneous tumors composed of luciferase-expressing MDA-MB-231 breast cancer cells. (**B**) Quantification of the photon flux from representative tumors demonstrates a large drop in bioluminescent signal by 6 hours. (**C**) 3-D micro-CT renderings of tumors (gray), and embedded vasculature (red), of the MDA-MB-231-Luc2 tumors. (**D**) Quantification of the binarized images demonstrating vessel volume (VV), and vessel density (VV/TV). Data represents averages ± s.e.m. (N = 3), asterisks indicate p<0.05.

To test the effectiveness of our analysis on a human cancer cell line, the responses of MDA-MB-231-Luc2 tumors were also analyzed. While residual bioluminescence was still present at 6 hours (**Figure 6A**), there was a significant drop (9-fold, p = 0.03) in total photon flux following DMXAA treatment (**Figure 6B**). At 24 hours, mice were terminally perfused with Microfil and tumors were excised and subjected to 3-D quantification. As with the NSCLC tumors, there was a decrease in vessel density in the MDA-MB-231 tumors of the DMXAA treated mice (**Figure 6C**). Similar to what was seen in the murine tumors, there was no significant difference in TV (data not shown), but a significant decrease was seen in both VV and VV/TV (**Figure 6D**) (p = 0.05 and p = 0.02, respectively). No significant difference was observed for V.N, V.Th, or V.Sp (data not shown). This data demonstrates the preclinical potential for such analyses to be applied to a wide variety of phenotypically diverse tumor lines, including assessments of their responses to therapeutic agents.

## Discussion

Improving upon 3-D methods of imaging tumor vasculature will enable a clearer understanding of the complexities of tumor angiogenesis, and responses to therapeutic intervention than can be provided by conventional 2-D histological methods. For this study, we selected Microfil, an agent that offers several advantages including: its wide use, ease of administration and tissue preparation, high-resolution of vasculature structure, long-term sample stability, and lack of extravasation [1], [29]. However, some of the drawbacks of this contrast agent include the inability for histological sectioning of samples [1], a similar x-ray contrast to bone [1], [29], and the inconsistent Microfil perfusion and x-ray absorption of the smaller capillary networks, that together with the inherent variations in contrast levels between capillaries and surrounding tissues, renders these networks difficult to resolve from the background ‘noise’ of the tumor itself [9]. As a result, small fragmented vessels may remain within the 3-D sample. The presence of these fragments may represent potential sources of error in some of the quantified indexes such as VV and VV/TV, however, as these events occur on the lower end of the scanning resolution (10 µm), the potential effect of these errors remains small. It would be possible to try and reconnect these fragmented vessels using computational methods that include expanding all vasculature until connections are made, and eroding back to original diameter, while maintaining the connections. However, since many vessels within the samples lie in close proximity to each other, this could lead to the creation of connections that were not actually present in the samples, thereby introducing additional errors into the measurements. The difficulties in resolving fine structural details, even at high-resolution micro-CT scanning, is a limitation that users must be aware of [9].

Herein, we have described the novel combination of Microfil with the maximal spheres analysis, for the simultaneous 3-D quantitative assessment of blood vessel microarchitecture and regions of non-perfusion. Using this technique it was possible to identify clear differences in tumor vessel structure and density that were characteristic and consistent within the various tumor lines evaluated. It is likely that the observed variations in vessel architecture amongst the different tumor models are reflective of their relative abilities to engage host cells in the angiogenic process. Indeed, it may be possible to establish correlations between angiogenesis patterns and pro-angiogenic factors that are released by different tumors. The observed tumor-specific differences in microvessel structure offers the novel potential for being able to correlate tumor-specific angiogenesis patterns with tumor progression, severity, and the responses to therapeutic intervention. Furthermore, studies assessing angiogenesis in humans with cancer using functional measures such as blood flow and blood volume have revealed correlations with therapeutic response [32], [33]. Since VV has been shown to be a relative measure of entire blood volume within the sample [21], this parameter could be used as a surrogate to assess the responses of tumors to novel agents.

Considerable efforts are currently underway to identify improved anti-angiogenic therapies for the control tumor growth [34]. There is thus an urgent need for experimental methods that can accurately quantify the effects of any given anti-angiogenic agent on different tumor types. As illustrated by our use of a VDA, the 3-D methods we employed provided a way to quantify, and visualize in 3-D, the impact of this anti-angiogenic agent. The differences in effectiveness between the two cell lines when given the DMXAA raises questions about the underlying reason(s) for this difference. The data shown herein, as well as results using other cell lines (data not shown), have indicated that total vessel volume and density show a strong correlation with the ability of an exogenously administered agent to access the tumor. Drug delivery via the aberrant tumor vasculature can be severely impeded as a result of tumors becoming progressively less well vascularised during their growth. As such, strategies for normalization of tumor vessels has become an important goal in cancer therapeutics [35]. Our system would allow a way to evaluate novel methods for vessel normalization by measuring the change in response to a given anti-angiogenic agent on poorly versus well vascularised tumors.

In addition, hypoxic and avascular regions within tumors are critically important not only with respect to issues concerning drug-delivery and response to ionizing radiation, but also in determining the effectiveness of hypoxia-activated drugs [36]. Hence, having the ability to accurately quantify pauci-vascular and avascular regions within tumors represents an important end-point parameter in preclinical assessments of treatment efficacy.

Lastly, the methods we describe to quantify ex-vivo blood vessel structure, as well as regions that are hypo- or avascular in tumors are readily applicable to other 3-D imaging techniques combined with micro-CT using alternate contrast agents such as iodine or barium sulphate [1], or to disease entities whose pathogenesis involves blood vessel pathology and angiogenesis, such as myocardial infarction, stroke, atherosclerosis, vasculitis, and inflammation.

## Supporting Information

Figure S1 **Micro-CT imaging of tumor vasculature.** X-ray micrograph (left) of a mouse bearing large metastases in the vicinity of the left pelvis (yellow box). 3-D rendering (right) of the Microfil-perfused metastasis reveals the tumor vasculature and general outline of the tumor (the left knee has been cropped out of picture). The sacrum (red asterisk) and iliac crest (yellow asterisk) are visible since Microfil has a similar contrast density to bone.
(TIF)

Figure S2 **Histology of necrotic areas in H1299 tumors.** H&E stained image of a H1299 subcutaneous tumor (left) showing largely empty necrotic areas (yellow arrowheads) in close proximity to vessels (black arrows) that would be predicted to be filled by the contrast medium. Immunohistochemical stain with a CD31-specific antibody (brown stain), and counterstained with hematoxylin, demonstrating small vessels (black arrows) in the proximity of an empty necrotic area (red arrowheads). The latter serving as sites of Microfil infiltration in the H1299-derived subcutaneous tumors.
(TIF)

Figure S3 **Histology of necrotic areas in 344SQ tumors.** Representative H&E stained images of a 344SQ-EL subcutaneous tumor showing extensive central necrosis.
(TIF)

Figure S4 **Micro-CT imaging of tumor necrosis.** To clearly visualize the extent of the avascular regions within a tumor, a demonstration of maximal-sphere filling model cutplanes through a subcutaneous tumor is shown through an entire sample (red indicates spheres that were 2 mm or greater in diameter).
(TIF)

Figure S5 **Histology of DMXAA treated tumors.** (**A**) Representative images of 344SQ-EL subcutaneous tumors 24 hrs post-treatment with DMXAA that were perfused with Microfil. The DMXAA-treated tumors were hemorrhagic, and lacked the vascular filling of the contrast medium that was seen with the control tumors. (**B**) Histologic images of subcutaneous tumors treated with DMXAA, or vehicle control, that had not been perfused with Microfil. H&E stained images demonstrate a haemorrhagic rim in the DMXAA treated tumors, with areas of ensuing necrosis underneath. Immunohistochemical staining with an alpha-smooth muscle actin-specific antibody (brown stain), counterstained with hematoxylin, demonstrating the dilated and clot-filled vessels in the tumor periphery (scale bars marked on images).
(TIF)

Figure S6 **Micro-CT quantification of DMXAA treated kidneys.** (**A**) 3-D renderings of kidneys (gray) with vasculature (green) from DMXAA-treated and vehicle-control mice. Quantification of vessel volume (VV) (**B**), vessel density (VV/TV) (**C**), vessel number (V.N) (**D**), vessel thickness (V.Th) (**E**), and vessel separation (V.Sp) (**F**), all demonstrate no significant difference between vehicle controls or DMXAA-treated mice.
(TIF)

Movie S1 **3-D rendering of tumor vasculature.** 3-D renderings of a Microfil-perfused subcutaneous tumor (344SQ cells) rotating around the central axis. Incremental images were used to generate the frames for the rotating 3-D movie for viewing. Tumor mass is represented in gray, vessels in red.
(MOV)

Movie S2 **3-D map of vessel thickness.** Vessel thickness heat map of a Microfil-perfused subcutaneous tumor (344SQ cells). Scale bar was changed in 10 µm diameter increments to generate images to use as frames to create the movie for viewing. Red scale changes from 10 µm diameter to 300 µm and back to 10 µm highlighting both the larger and smaller vessels.
(MOV)

Movie S3 **3-D maximal spheres model of avascular/necrotic regions.** 3-D renderings of the maximal spheres model of vessel separation. Red indicates a 2 mm, or greater sphere diameter. Images were generated to represent the intact specimens, followed by incremental cutplanes through the samples (20 cutplanes per sample) to create frames for a movie for viewing. Two representative tumor samples showing both the heterogeneity and extent of the avascular areas as compared to the kidney control on the left.
(MOV)
