# Supplementary figures and images for: Quantitative Ex-Vivo Micro-Computed Tomographic Imaging of Blood Vessels and Necrotic Regions within Tumors
Source: PLoS One. 2012 Jul 25;7(7):e41685. doi: 10.1371/journal.pone.0041685 (PMC3404997; doi:10.1371/journal.pone.0041685)

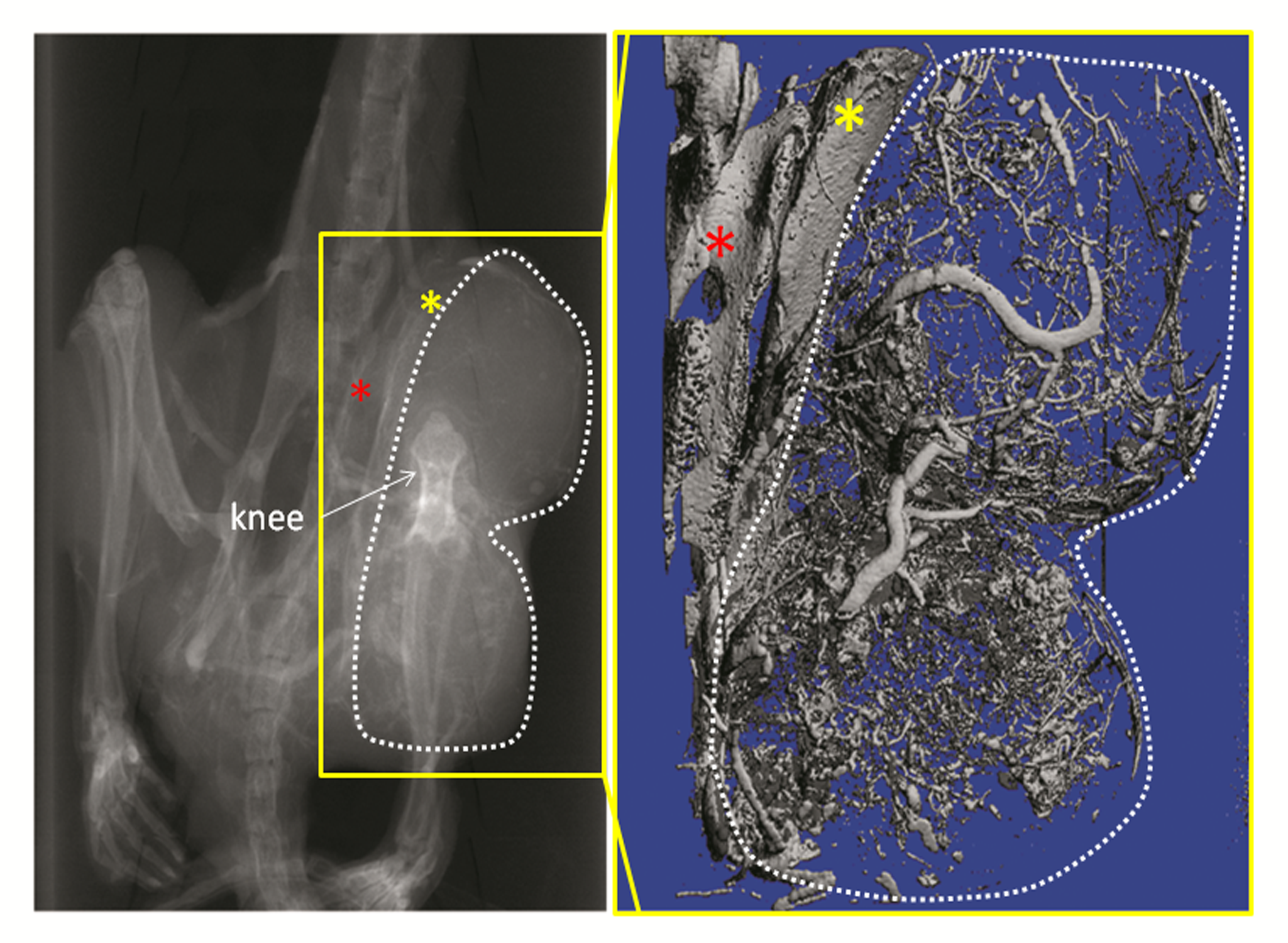

Supplement: Figure S1 — Micro-CT imaging of tumor vasculature. X-ray micrograph (left) of a mouse bearing large metastases in the vicinity of the left pelvis (yellow box). 3-D rendering (right) of the Microfil-perfused metastasis reveals the tumor vasculature and general outline of the tumor (the left knee has been cropped out of picture). The sacrum (red asterisk) and iliac crest (yellow asterisk) are visible since Microfil has a similar contrast density to bone. (TIF) [file pone.0041685.s001.tif]

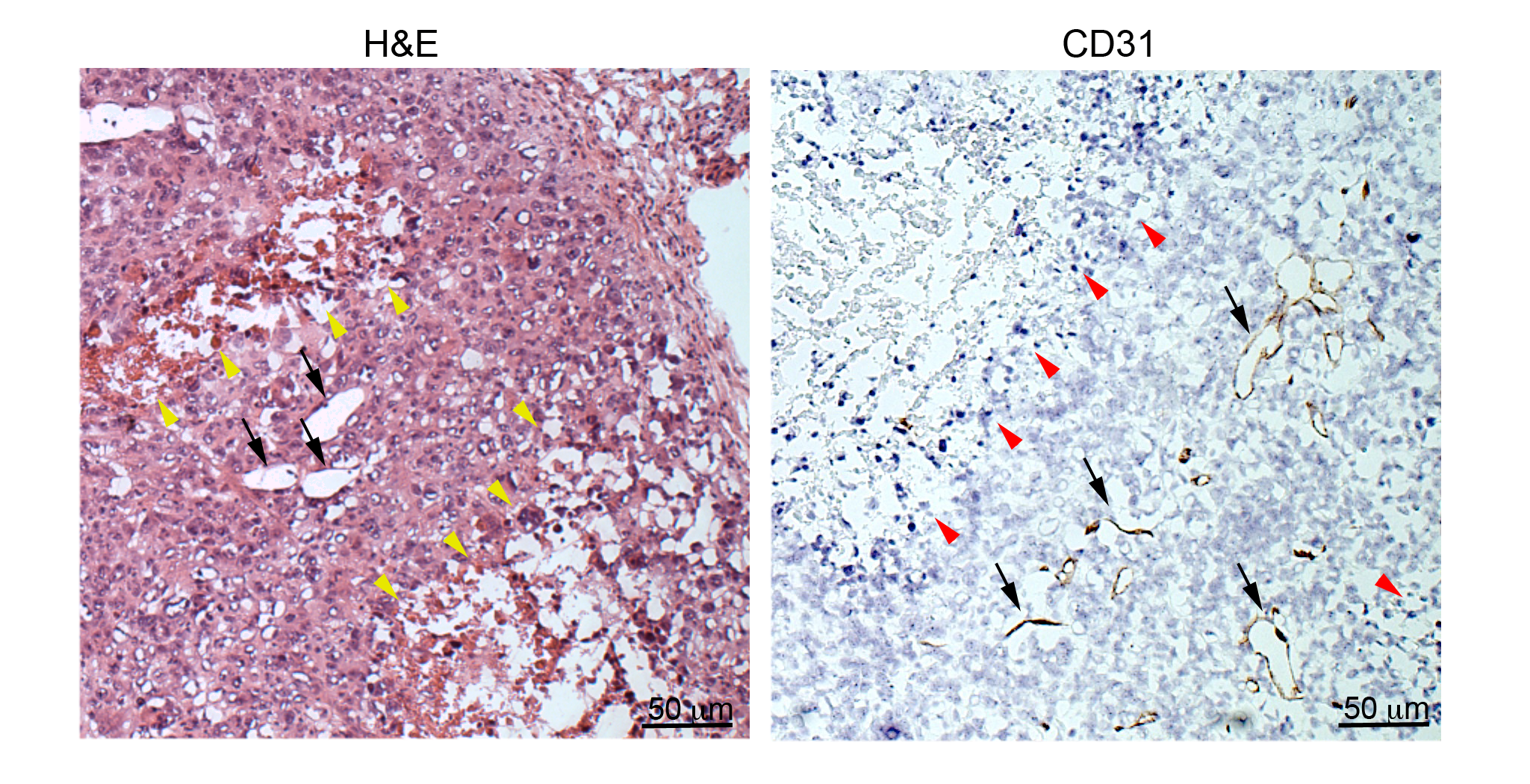

Supplement: Figure S2 — Histology of necrotic areas in H1299 tumors. H&E stained image of a H1299 subcutaneous tumor (left) showing largely empty necrotic areas (yellow arrowheads) in close proximity to vessels (black arrows) that would be predicted to be filled by the contrast medium. Immunohistochemical stain with a CD31-specific antibody (brown stain), and counterstained with hematoxylin, demonstrating small vessels (black arrows) in the proximity of an empty necrotic area (red arrowheads). The latter serving as sites of Microfil infiltration in the H1299-derived subcutaneous tumors. (TIF) [file pone.0041685.s002.tif]

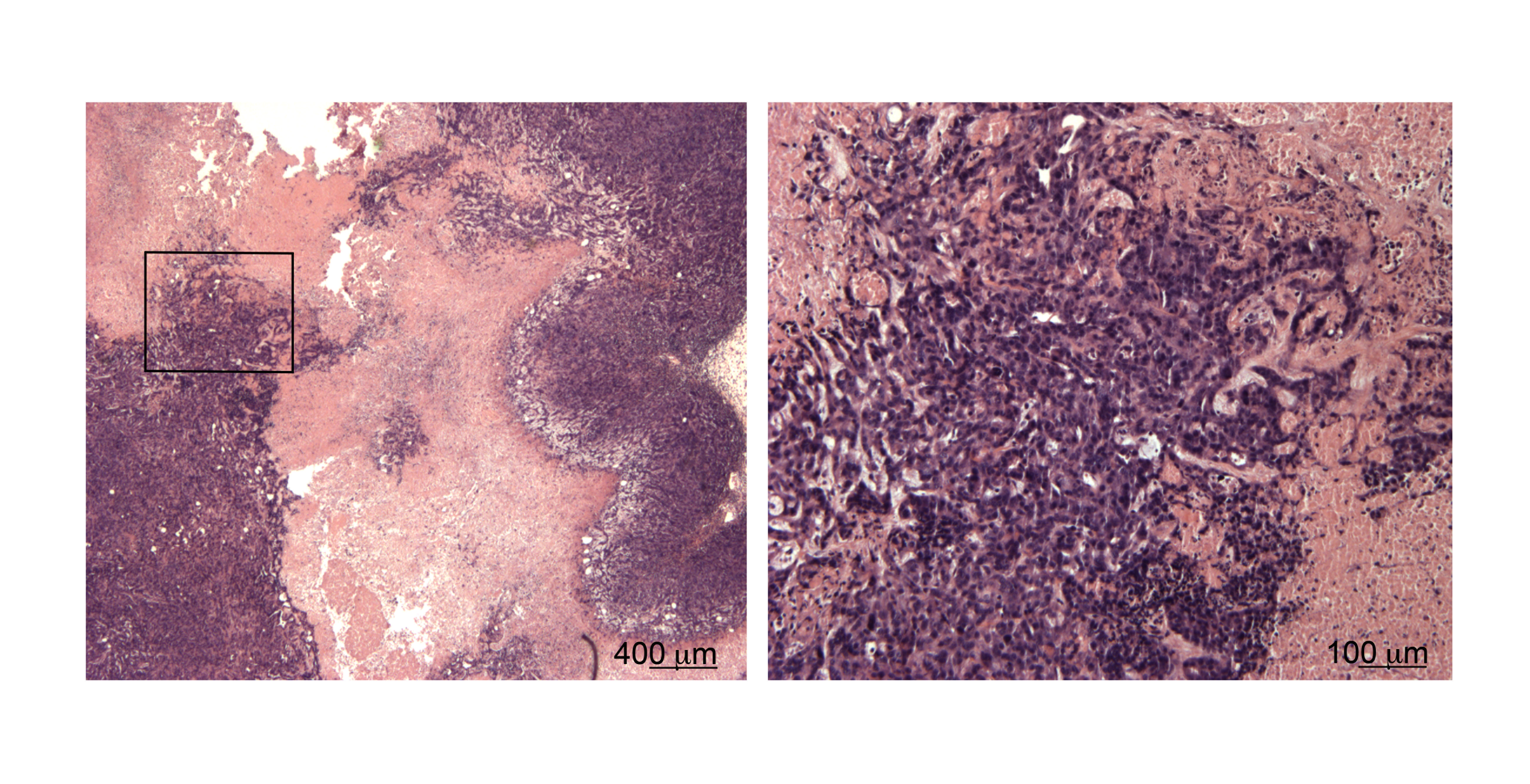

Supplement: Figure S3 — Histology of necrotic areas in 344SQ tumors. Representative H&E stained images of a 344SQ-EL subcutaneous tumor showing extensive central necrosis. (TIF) [file pone.0041685.s003.tif]

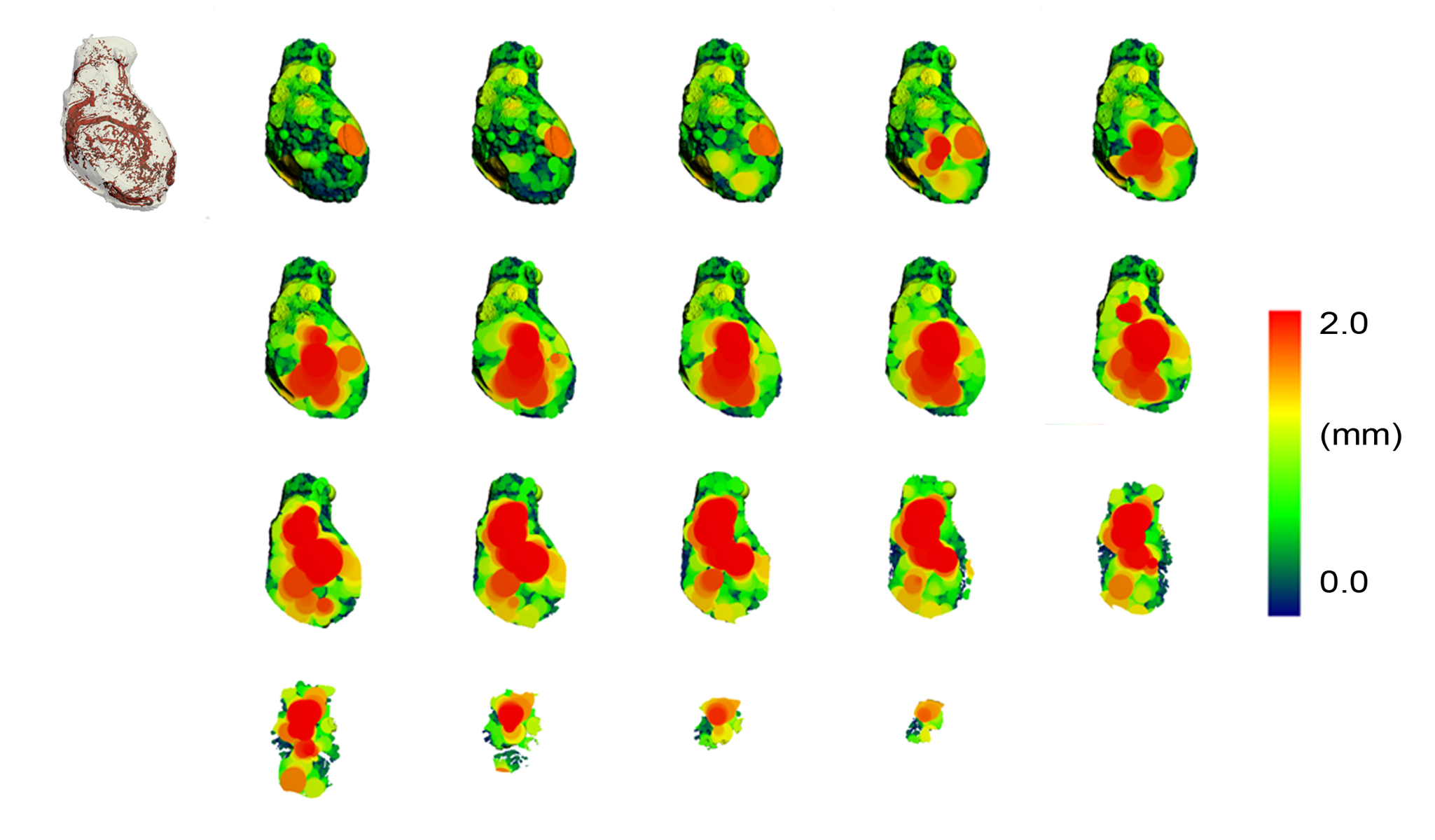

Supplement: Figure S4 — Micro-CT imaging of tumor necrosis. To clearly visualize the extent of the avascular regions within a tumor, a demonstration of maximal-sphere filling model cutplanes through a subcutaneous tumor is shown through an entire sample (red indicates spheres that were 2 mm or greater in diameter). (TIF) [file pone.0041685.s004.tif]

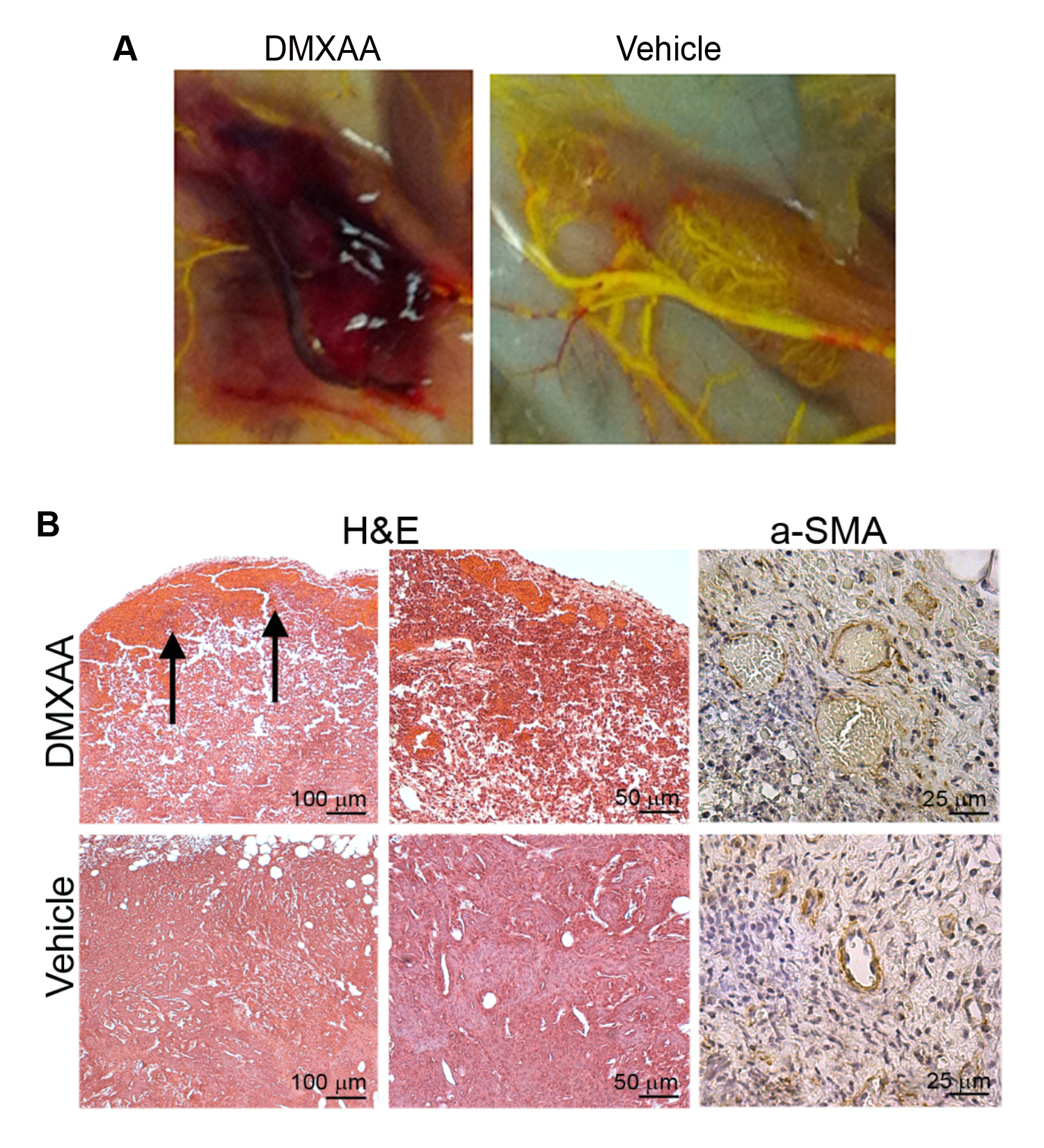

Supplement: Figure S5 — Histology of DMXAA treated tumors. (A) Representative images of 344SQ-EL subcutaneous tumors 24 hrs post-treatment with DMXAA that were perfused with Microfil. The DMXAA-treated tumors were hemorrhagic, and lacked the vascular filling of the contrast medium that was seen with the control tumors. (B) Histologic images of subcutaneous tumors treated with DMXAA, or vehicle control, that had not been perfused with Microfil. H&E stained images demonstrate a haemorrhagic rim in the DMXAA treated tumors, with areas of ensuing necrosis underneath. Immunohistochemical staining with an alpha-smooth muscle actin-specific antibody (brown stain), counterstained with hematoxylin, demonstrating the dilated and clot-filled vessels in the tumor periphery (scale bars marked on images). (TIF) [file pone.0041685.s005.tif]

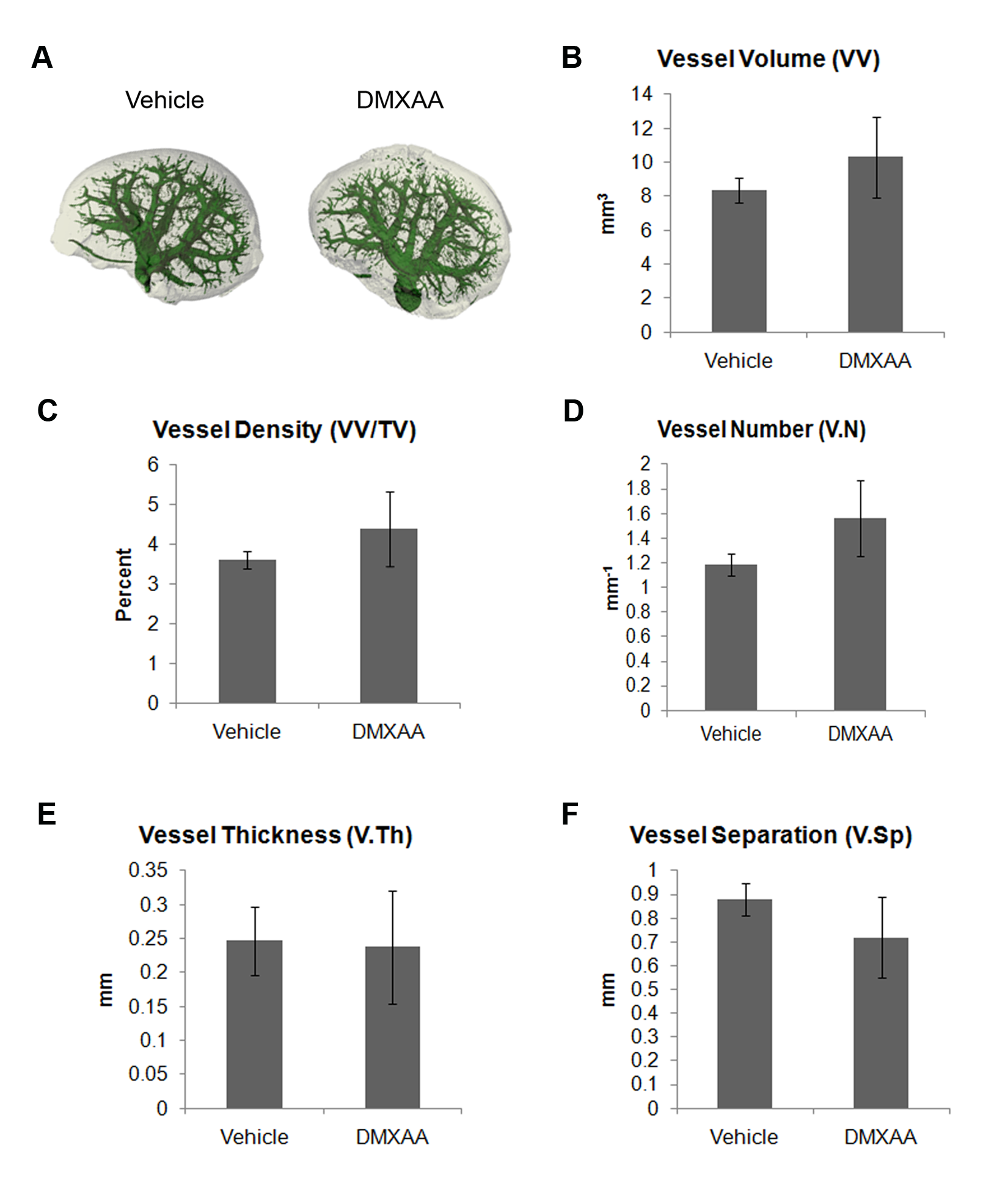

Supplement: Figure S6 — Micro-CT quantification of DMXAA treated kidneys. (A) 3-D renderings of kidneys (gray) with vasculature (green) from DMXAA-treated and vehicle-control mice. Quantification of vessel volume (VV) (B), vessel density (VV/TV) (C), vessel number (V.N) (D), vessel thickness (V.Th) (E), and vessel separation (V.Sp) (F), all demonstrate no significant difference between vehicle controls or DMXAA-treated mice. (TIF) [file pone.0041685.s006.tif]
